# Status and factors related to post‐traumatic growth in continuous ambulatory peritoneal dialysis: A multi‐centre study

**DOI:** 10.1002/nop2.1096

**Published:** 2021-10-17

**Authors:** Xing Li, Xueli Zhou, Dengyan Ma, Stephen Salerno, Min Qi, Yongshu Diao, Chongcheng Chen, Hui Zhong, Shuxin Xiao, Yan Wang, Jiaju Zhang, Hongyan Luo, Lijun Huang, Santao Ou, Yuanmei Li, Xiaoyun Cheng, Huihui Xu, Yan Ma, Yi Li, Jianing Wei

**Affiliations:** ^1^ Nephrology Department of West China Hospital School of Nursing Sichuan University Chengdu China; ^2^ Department of Biostatistics University of Michigan Ann Arbor Michigan USA; ^3^ Nephrology Department of the Third People's Hospital Chengdu China; ^4^ Nephrology Department of the Peoples' Hospital Meishan China; ^5^ Nephrology Department of Central Hospital Panzhihua China; ^6^ Nephrology Department of People's Hospital Ningxia Autonomous Region Yinchuan China; ^7^ Nephrology Department of the Peoples' Hospital Jianyang China; ^8^ Nephrology Department of Affiliated Hospital Southwest Medical University Luzhou China; ^9^ Nephrology Department of Central Hospital Mianyang China; ^10^ Nephrology Department of the Second Peoples' Hospital Chengdu China; ^11^ Nephrology Department of the First people's Hospital Jiujiang China; ^12^ Nephrology Department of the First people's Hospital Liangshan China

**Keywords:** peritoneal dialysis, post‐traumatic growth, psychological experience, self‐perceived burden, self‐perceived stress

## Abstract

**Aim:**

To investigate the extent of post‐traumatic growth, and the correlation between post‐traumatic growth and self‐perceived stress, post‐traumatic growth and self‐perceived burden among CAPD patients.

**Design:**

A cross‐sectional study.

**Methods:**

This was a multi‐centre study including 752 patients from 44 hospitals. Self‐perceived stress, self‐perceived burden and post‐traumatic growth were measured using the post‐traumatic growth inventory (PTGI), the Chinese version of the perceived stress questionnaire (CPSQ) and the self‐perceived burden scale (SPBS). A multiple stepwise regression analysis was fit with the total PTGI score as the outcome of interest.

**Results:**

Patients concurrently experienced post‐traumatic growth and stress following peritoneal dialysis. The initiation of patients’ education level, employment status and self‐perceived stress were all found to relate to growth among Chinese CAPD patients. There was not sufficient evidence to suggest that self‐perceived burden was related to experiencing growth.

## 1 INTRODUCTION

Peritoneal dialysis (PD) is a renal replacement therapy entails the dwelling of dialysis fluid in the peritoneal cavity for a period of time, during which time solutes, fluid and uraemic metabolites pass from peritoneal capillaries, across the peritoneal membrane, into the peritoneal fluid by the process of diffusion and osmosis. Types of PD include intermittent peritoneal dialysis, continuous cycling PD and continuous ambulatory peritoneal dialysis (CAPD). CAPD, a method of uninterrupted dialysis, is one of the main treatment options for end‐stage renal disease (Lu et al., 2020). Although CAPD is effective in eliminating metabolic waste and correcting acid, alkali, water and electrolyte‐related disorders, it often disrupts daily routines, hinders quality of life and elicits a range of emotional experiences (Ruiz et al., 2017). These emotional experiences, however, may lead to positive psychological changes, namely post‐traumatic growth (Boyle et al., 2017).

## 2 BACKGROUND

Post‐traumatic growth refers to the positive changes an individual undergoes after a traumatic event, such as a natural disaster or illness (Tedeschi & Calhoun, 1996). It has been reported that post‐traumatic growth is associated with disease status and psychological condition (Casellas‐Grau et al., 2017; Koutná et al., 2017). Several studies have shown that patients with more post‐traumatic growth are more likely to adopt positive coping strategies and have less painful future experiences (Garnefski et al., 2008). Additionally, post‐traumatic growth promotes active, healthy behaviour in cancer patients (Lim, 2019). Despite evidence that post‐traumatic growth broadly alleviates negative psychological experiences and promotes disease recovery, most research focuses on cancer patients or victims of natural disasters. Few studies have examined post‐traumatic growth among PD patients and are limited to the relationship between post‐traumatic growth, social support and coping styles (Casellas‐Grau et al., 2017).

Stress and psychological burden are common experiences for PD patients (Makhija et al., 2017). While there may be a correlation between self‐perceived stress, self‐perceived burden and post‐traumatic growth (Wu et al., 2019), the relationship amongst these variables in PD patients has rarely been reported in the literature. This study will investigate post‐traumatic growth in CAPD patients across multiple centres and analyse its relationship with self‐perceived stress and self‐perceived burden to further supplement the existing literature on post‐traumatic growth in PD patients and its influencing factors.

## 3 METHODS

### 3.1 Design and participants

A cross‐sectional study was carried out to examine the relationship between post‐traumatic growth, self‐perceived stress and self‐perceived burden among CAPD patients. Study participants were patients who had initiated or were currently undergoing continuous ambulatory peritoneal dialysis in the nephrology units of 44 hospitals between July and October 2019. Inclusion criteria were (a) age over 18 years, (b) physical, mental and linguistic capacity to answer the questionnaire items, as assessed by the research nurse gathering the data and (c) provision of informed consent. Exclusion criteria were (a) severe cognitive impairment, as assessed by the nurse gathering the data, (b) functional or organic mental disease, (c) uraemia complications with serious infection or injury to the cardiopulmonary organs or (d) receipt of haemodialysis or continuous renal replacement in the past three months.

### 3.2 Instruments

#### 3.2.1 Socio‐demographic characteristics form

In an initial assessment, patient age, sex, educational level, living status, employment status, marital status, family income per capita, insurance status, disease status, the time since initiated PD, whether the patient changes dialysis fluid on their own, presence of dialysis‐related comorbidities (peritonitis, catheter dysfunction, tunnel opening infection, peritoneal fluid leakage, hernia, pulmonary infection, electrolyte disturbance and others) and whether or not the patient had children were collected. Furthermore, the following instruments were administered.

##### Post‐traumatic growth inventory

The post‐traumatic growth inventory (PTGI) reports changes in all five dimensions of post‐traumatic growth: (1) new possibilities, (2) personal strengths, (3) relatedness to others, (4) spiritual changes and (5) appreciation for life. The PGTI is comprised of 20 items, each on a six‐point Likert scale from 0 (no change as a result of the event) to 5 (the highest degree of change). A total PTGI score is calculated as the sum of these individual items, ranging from 0–100, with higher scores indicating more growth. The Chinese version of the PTGI has been shown to demonstrate strong internal consistency (Cronbach's α = 0.867) and test–retest reliability in other samples (*r* = 0.705; Geng et al., 2011).

##### Chinese version of the perceived stress questionnaire

The Chinese version of the perceived stress questionnaire (CPSQ), with 30 items, asks how often each of the listed feelings or thoughts occurred (Luo et al., 2018) and has shown satisfactory validity and reliability (Cronbach's α = 0.922, *r* = 0.782) (Luo et al., 2018; Meng et al., 2020). The items are scored on a four‐point Likert scale from 1 (almost never) to 4 (usually), and the total CPSQ score ranges from 30–120. A CPSQ Index is defined by subtracting 30 from the total score and dividing by 90. The resulting index ranges from 0–1, and higher values indicate higher levels of stress. The index is then divided into four classes (lower, moderate, severe and extreme) with cut‐off values of 0.25, 0.35 and 0.45.

##### Self‐perceived burden scale

The Self‐perceived burden scale (SPBS) consists of ten items, including three dimensions of bodily, emotional and economic burden. Each item is scored on a five‐point Likert scale, from 1 (never) to 5 (always). Total SPBS score is defined as the sum of the individual items, and the eighth item is scored in reverse. The SPBS score was classified into four groups (no significant, mild, moderate or severe self‐perceived burden), with critical values at 20, 30 and 40. The SPBS has strong internal consistency (Cronbach's α = 0.85) (Natalie et al., 2003), and convergent validity of SPBS was demonstrated with global quality of life (*r* = −0.546), physical well‐being (*r* = −0.547), emotional well‐being (*r* = −0.549), functional well‐being (*r* = −0.404), financial satisfaction (*r* = −0.284) and depression (*r* = 0.414; Simmons, 2007).

### 3.3 Data collection

Data were collected for 3 months between July and October 2019. After obtaining the corresponding permissions from the research units and ethics committees, nine research nurses were selected and each was assigned to a dialysis unit. The nurses were trained at the same time to ensure homogeneity in the procedure. During data collection, six coordination meetings were held. Each nurse explained to their patients with an information letter explaining the purpose of the study and information related to anonymity and voluntary participation. Patients completed the questionnaires with the help of the nurses. The following variables were recorded as follows: age, sex, educational level, living status, employment status, marital status, family income per capita, insurance status, disease status, dialysis vintage, dialysate replacement method, comorbidities of dialysis, whether have children, PTGI, CPSQ and SPBS. 752 patients were recruited, and questionnaires were collected on the spot by online filling. The online questionnaire is designed to be submitted only after each item is filled out. Therefore, the response rate of the questionnaire is 100%.

### 3.4 Ethical considerations and procedures

Review board approval was obtained from the ethics committees of the participating hospitals in September 2019 (NO. 2019/715). Participants received written information about study aims, voluntary and anonymous participation. Implied informed consent was considered by returning the questionnaire completed.

### 3.5 Statistical analysis

Data analysis was performed in IBM SPSS, version 22 (IBM Corp. Released 2013. IBM SPSS Statistics for Windows, Version 22.0, Armon, NY: IBM Corp.) Descriptive statistics for the participant's characteristics, as well as their scores on the various instruments, were reported as frequencies and percentages or means and standard deviations. Independent *t* test, *F*‐tests and Pearson's correlations were calculated to examine the unadjusted associations of these characteristics, self‐perceived stress and self‐perceived burden, with total PTGI score. Next, a multiple stepwise regression analysis was carried out to determine whether significant associations persisted. Categorical variables were coded with dummy variables. Variables related to total PTGI score were taken into the model. A significance level of .05 was taken to determine whether any statistically significant relationships existed in the hierarchical analysis.

## 4 RESULTS

### 4.1 Sample characteristics and bivariate associations

As shown, 752 patients were recruited into the study, and largest group came from the West China Hospital of Sichuan University (*n* = 289, 38.4%). Socio‐demographic and clinical characteristics for the participants are presented in Table 1. Most patients were married (81.2%), not working (78.1%), have had kidney disease for at least 36 months (64.1%) and have relied on dialysis for at least 12 months (65.9%). Roughly half were female (49.4%), and a minority (27.9%) had dialysis‐related complications. Table 1 shows that most participants were of working age. The average total PTGI score in the study was 57.36 ± 15.841 out of a maximum possible score of 100. Cronbach's α for the study's PTGI was 0.917. Similarly, Cronbach's α for the CPSQ was 0.845 and 0.924 for the SPBS.

**TABLE 1:** Socio‐demographic and clinical characteristics of the *n* = 752 study participants

Variable	Number	Count (%)	PTGI‐Total Score (Mean ± *SD*)	*r/F/t*	*p*
Age, (mean ± *SD*)	46.71 ± 12.737^a^	57.36 ± 15.841	0.034^c^	0.348	
Sex					
Female	371	49.4%	57.25 ± 16.673^b^	−0.190^d^	.849
Male	381	50.6%	57.47 ± 14.962 ^b^		
Educational level					
Primary school	150	19.9%	60.91 ± 15.865^b^	3.860^e^	.009
Junior high school	274	36.5%	55.48 ± 16.206^b^		
Senior high school	147	19.6%	57.07 ± 16.538^b^		
Junior college or above	181	24.0%	57.50 ± 14.232^b^		
Living status					
Lives alone	58	7.7%	59.57 ± 14.678^b^	1.105^d^	.269
Does not live alone	694	92.3%	57.18 ± 15.931^b^		
Employment status					
Employed	165	21.9%	60.35 ± 15.210^b^	2.757^d^	.006
Not employed	587	78.1%	56.52 ± 15.926^b^		
Marital status					
Single	84	11.2%	54.56 ± 17.564^b^	1.692^e^	.167
Married	611	81.2%	57.70 ± 15.757^b^		
Divorced	40	5.3%	59.83 ± 12.287^b^		
Widowed	17	2.3%	53.18 ± 16.284^b^		
Family income per capita (¥)					
Less than 3,000	449	59.7%	57.41 ± 15.620^b^	0.177^e^	.912
At least 3,000	189	25.1%	57.49 ± 16.265^b^		
At least 5,000	64	8.5%	56.08 ± 14.778^b^		
At least 8,000	50	6.7%	58.06 ± 17.806^b^		
Insurance status					
Government insurance	7	0.9%	64.57 ± 21.793^b^	1.368^e^	.255
Medical insurance/social security/commercial insurance	701	93.2%	57.46 ± 15.723^b^		
Out of pocket/no insurance	44	5.9%	54.68 ± 16.616^b^		
Disease status					
Less than 6 months	55	7.3%	58.64 ± 13.235^b^	1.357^e^	.255
At least 6 months	69	9.2%	54.41 ± 19.985^b^		
At least 12 months	146	19.4%	58.83 ± 15.828^b^		
At least 36 months	482	64.1%	57.19 ± 15.841^b^		
Dialysis vintage					
Less than a month	32	4.2%	59.09 ± 17.030^b^	0.265^e^	.900
At least a month	49	6.5%	58.55 ± 17.372^b^		
At least 4 months	79	10.5%	58.14 ± 20.034^b^		
At least 7 months	97	12.9%	57.19 ± 15.913^b^		
At least 12 months	495	65.9%	57.04 ± 14.861^b^		
Dialysate replacement method					
Yes	662	88%	57.34 ± 15.481^b^	−0.072^d^	.943
No	90	12%	57.49 ± 18.375^b^		
Comorbidities of dialysis					
Yes	210	27.9%	56.50 ± 14.817^b^	0.922^d^	.357
No	542	72.1%	57.69 ± 16.222^b^		
Have children					
Yes	639	85%	57.56 ± 15.573^b^	−0.842^d^	.400
No	113	15%	56.20 ± 17.305^b^		

Notes

Frequencies and percentages for categorical variables are presented in addition to the mean ± *SD* total PTGI score within each factor level. Unadjusted associations via *F* or *t* tests, the requisite test statistics and the *p*‐values reported. a = Age; b = PTGI‐Total Score; c = Pearson correlation (*r*); d = *t*‐statistic, e = *F*‐statistic.

The results of the CPSQ show that 8.9% of participants had lower, 28.6% had moderate, 36.7% had severe, and 25.8% had extreme self‐perceived stress. Self‐perceived burden was shown to be primarily mild or severe, accounting for 39.8% and 31.1% of the study sample, respectively. Contrary to previous studies, age, sex and marital status were not significantly associated with post‐traumatic growth in this study population. However, patients who were working reported significantly more growth than those who were not (*p* = .006). Correlations among the main study variables are reported in Table 2. Although self‐perceived stress (total CPSQ score) was found to be positively correlated with post‐traumatic growth (*p* = .001), the correlation coefficient was relatively small in magnitude (*r* = 0.125). Total self‐perceived burden score was not found to be associated with total PTGI score; however, self‐perceived burden was associated with the new possibility dimension of the PTGI scale (*p* = .012).

**TABLE 2:** Pairwise Pearson correlations between PTGI, CPSQ and SPBS survey instrument responses

Variables/Mean ± *SD*	1	2	3	4	5	6	7	8	9	10	11	12	13	14	15	16
1. CPSQ: Conflict	1.000															
2. CPSQ: Overload	0.554^a^	1.000														
3. CPSQ: Joy	−0.341^a^	−0.171^a^	1.000													
4. CPSQ: Worries/Tension	0.746^a^	0.606^a^	−0.437^a^	1.000												
5. CPSQ: Self‐Realization	0.157^a^	0.348^a^	0.300^a^	0.161^a^	1.000											
6. CPSQ: Total Score	0.770^a^	0.747^a^	0.010^c^	0.852^a^	0.459^a^	1.000										
7. SPBS: Body Burden	0.370^a^	0.306^a^	−0.209^a^	0.493^a^	0.116^a^	0.429^a^	1.000									
8. SPBS: Financial Burden	0.325^a^	0.234^a^	−0.209^a^	0.463^a^	0.057 ^c^	0.375^a^	0.679^a^	1.000								
9. SPBS: Emotional Burden	0.452^a^	0.342^a^	−0.220^a^	0.582^a^	0.141^a^	0.517^a^	0.827^a^	0.689^a^	1.000							
10. SPBS: Total Score	0.430^a^	0.336^a^	−0.230^a^	0.568^a^	0.128^a^	0.495^a^	0.950^a^	0.784^a^	0.951^a^	1.000						
11. PTGI: Total Score	−0.107^a^	0.131^a^	0.484^a^	−0.155^a^	0.319^a^	0.125^a^	−0.008^c^	−0.037^c^	−0.008^c^	−0.013^c^	1.000					
12. PTGI: Relating to Others	−0.126^a^	0.110^a^	0.434^a^	−0.163^a^	0.261^a^	0.083^b^	<0.001^c^	<0.001^c^	−0.013^c^	−0.006^c^	0.916^a^	1.000				
13. PTGI: New Possibilities	−0.200^a^	0.068^c^	0.480^a^	−0.236^a^	0.253^a^	0.023^c^	−0.071^c^	−0.090^b^	−0.097^a^	−0.091^b^	0.788^a^	0.734^a^	1.000			
14. PTGI: Personal Strength	−0.050^c^	0.016^c^	0.400^a^	−0.072^b^	0.203^a^	0.124^a^	−0.042^c^	−0.069^c^	−0.020^c^	−0.039^c^	0.854^a^	0.689^a^	0.579^a^	1.000		
15. PTGI: Spiritual Change	−0.050^c^	0.016^c^	0.400^a^	−0.072^b^	0.203^a^	0.124^a^	0.019^c^	−0.003^c^	0.045^c^	0.029^c^	0.722^a^	0.559^a^	0.421^a^	0.620^a^	1.000	
16. PTGI: Appreciation for Life	0.021^c^	0.229^a^	0.261^a^	<0.001^c^	0.278^a^	0.191^a^	0.049^c^	−0.007^c^	0.042^c^	0.041^c^	0.790^a^	0.674^a^	0.554^a^	0.562^a^	0.426^a^	1.000
Mean ± *SD*	9.58 ± 2.776	8.92 ± 2.373	17.63 ± 4.341	24.49 ± 7.426	4.51 ± 1.211	65.12 ± 10.836	14.65 ± 4.195	2.98 ± 1.330	10.69 ± 4.173	28.32 ± 8.998	57.36 ± 15.841	19.20 ± 5.340	9.74 ± 2.739	9.84 ± 4.296	8.29 ± 3.065	10.29 ± 3.684

Notes

a = *p* < .01; b = *p* < .05; c = *p* > .05.

We use red for positive correlation and blue for negative correlation, and the color increases with the degree of correlation.

### 4.2 Multiple stepwise regression analysis of total PTGI score

In the regression, categorical variables are encoded as dummy variables. Multiple stepwise regression analyses showed that except for CPSQ conflict score and CPSQ worried/tension score, on model six educational level (*p* = .001), employment status (*p* = .009), CPSQ overload score (*p* = .000), CPSQ joy score (*p* = .000), CPSQ self‐realization score (*p* = .000) and CPSQ total score (*p* = .004), which were all significantly associated with post‐traumatic growth. These predictors explained 30.7% of the variation in post‐traumatic growth among the study participants (*F* = 56.561, *p* = .000) (Table 3).

**TABLE 3:** Factors related to post‐traumatic growth in the multiple stepwise regression analysis on *n* = 752 study participants

Variable	*B*	*SE*	*β*	*t*	*R^2^*	*p/F*
Model 1					0.234	229.983^a^
(Normal)	26.202	2.116	–	12.383		.000^b^
Joy	1.768	0.117	0.484	15.165		.000^b^
Model 2					0.280	147.030^a^
(Normal)	10.640	3.020		3.523		.000^b^
Joy	1.905	0.115	0.522	16.615		.000^b^
CPSQ: Overload	1.473	0.210	0.221	7.020		.000^b^
Model 3					0.289	102.666^a^
(Normal)	9.425	3.025		3.115		.002^b^
Joy	1.751	0.124	0.480	14.152		.000^b^
CPSQ: Overload	1.159	0.230	0.174	5.031		.000^b^
CPSQ: Self‐Realization	1.495	0.466	0.114	3.208		.001^b^
Model 4					0.298	80.605^a^
(Normal)	15.439	3.533		4.370		.000^b^
Joy	1.790	0.124	0.491	14.492		.000^b^
CPSQ: Overload	1.887	0.321	0.283	5.884		.000^b^
CPSQ: Self‐Realization	1.904	0.480	0.146	3.967		.000^b^
CPSQ: Total Score	−0.231	0.071	−0.158	−3.241		.001^b^
Model 5					0.302	66.009^a^
(Normal)	17.841	3.664		4.869		.000^b^
Joy	1.807	0.123	0.495	14.647		.000^b^
CPSQ: Overload	1.905	0.320	0.285	5.957		.000^b^
CPSQ: Self‐Realization	1.904	0.479	0.146	3.980		.000^b^
CPSQ: Total Score	−0.234	0.071	−0.160	−3.294		.001^b^
Educational Level	−1.069	0.451	−0.072	−2.372		.018^b^
Model 6					0.307	56.561^a^
(Normal)	25.257	4.631		5.454		.000^b^
Joy	1.805	0.123	0.495	14.691		.000^b^
CPSQ: Overload	1.759	0.323	0.263	5.439		.000^b^
CPSQ: Self‐Realization	1.774	0.479	0.136	3.702		.000^b^
CPSQ: Total Score	−0.207	0.072	−0.142	−2.897		.004^b^
Educational Level	−1.575	0.489	−0.107	−3.219		.001^b^
Employment Status	−3.365	1.293	−0.088	−2.602		.009^b^

Note

a = *F*; b = *p*.

Abbreviation: *B*, Unstandardized regression coefficients; *SE*, Standard error; *β*, Standardized regression coefficients; *t*, *t*‐statistic; *R^2^*, Adjusted *R^2^*; and *p*, *p*‐value.

## 5 DISCUSSION

This study showed that, although PD is extremely stressful, a majority of patients experienced positive post‐traumatic growth. This finding was consistent with studies in other disease populations, suggesting that PD patients are similar in their ability to garner positive growth from their experiences. However, we noted that the estimated PTGI scores were different for the other disease populations and across multiple studies, for example, reported a mean score of 50.72 in a group of type II diabetes patients (Purc‐Stephenson, 2014), while estimated a mean score of 61.15 among cancer survivors (Zhang, Lu, et al., 2020). Higher versus lower scores may be related to disease‐specific characteristics. In this study, the mean score of PTGI is 57.36 in CAPD patient. CAPD patients may exhibit more stress than diabetics, changing their peritoneal dialysate 2 to 4 times a day, and retaining dialysate in their abdominal cavity for 4 to 6 hr during the day (Maharjan et al., 2019). Kidney disease is a chronic disease, compared with cancers, and it is linked to less disability and pain. Patients with kidney disease showed lower or higher levels of distress than other diseases, which might influence their perception of growth.

We found that education level and employment status were two socio‐demographic factors associated with post‐traumatic growth. Specifically, patients with primary school education showed higher levels of PTG than junior high school, senior high school and junior college or above education. Those with primary school education enter social work at an earlier age and have more social experience, which may help them cope with the stress of illness. We also found that patients with senior high school and junior college or above education had higher PTG levels than those with junior high school. This may be because patients with senior high school and junior college or above education have more access to information related to their disease, and this may affect their outlook and perceptions. We also found that growth levels were higher among employed patients. It is possible that employment provides another source of spiritual and material contact with colleagues. This contact can foster additional support from others and have a positive emotional effect.

The main objective of this study was to examine the association between self‐perceived stress, self‐perceived burden and post‐traumatic growth in kidney disease patients undergoing continuous ambulatory peritoneal dialysis. We found that self‐perceived stress (total CPSQ score) was positively correlated with post‐traumatic growth (*p* = .001), but the correlation coefficient was relatively small in magnitude (*r* = 0.125). It indicates that perceived stress may promote post‐traumatic growth. This finding is consistent with previous studies in the field. Groarke et al. did find some evidence to support the hypothesis that stress is related to higher post‐traumatic growth, specifically greater cancer‐specific stress at diagnosis predicted higher post‐traumatic growth 6 months later (Groarke et al., 2017). Other studies also reported a positive relationship between stress caused by illness and post‐traumatic growth over time. But the results of some studies are inconsistent with our results (Bellizzi & Blank, 2006; Mcdonough et al., 2014). Ruiz et al. reported that low‐growth patients scored lower on stress metrics than the patients with decreasing or high growth (Ruiz et al., 2017). Mindfulness‐based stress reduction programmes have also been shown to increase growth among breast cancer survivors in China (Zhang et al., 2017). Stress reduction programmes help patients to maintain a calm state. The mechanism by which these programmes act on post‐traumatic growth may be related to how patients process their experiences. However, the participants in the present study were different from those in these studies. It may be that after CAPD, patients are better able to appreciate their personal health, thereby experiencing bearable stress and more growth. And this fits with theoretical models suggesting that a traumatic event precipitates cognitive processing that triggers attempts to establish meaning in response to the event (Janoff‐Bulman, 1989). Findings in the present study extend previous research and provide some additional support for the idea that struggle with a challenging illness may be instrumental in facilitating positive growth.

Unexpectedly, we did not find an association between self‐perceived burden and post‐traumatic growth. However, this finding can help to narrow the pathways guiding future research on this topic. There is little research on self‐perceived burden and post‐traumatic growth. To our knowledge, there is only one study on this relationship, which found only a weak correlation (*r* = −0.21) (Zhang, Gao, et al., 2019). The mechanism, by which self‐perceived burden influences growth, may be through patients’ resilience (Li et al., 2018). Resilience can be affected by multiple factors, including social‐emotional support and personal cognition (Southwick & Charney, 2012). Therefore, further study is necessary to elucidate the relationship between self‐perceived burden and post‐traumatic growth.

This study has several limitations. Firstly, as a cross‐sectional study, a causal relationship cannot be inferred. The study cannot provide information on dynamic changes in growth after PD over time. Secondly, the effect of the treatment method on patients’ psychology was not considered. It will be necessary to perform follow‐up assessments from the CAPD catheterization of kidney disease patients, analyse dynamic changes in post‐traumatic growth levels in various time periods and comprehensively integrate the relevant factors of post‐traumatic growth to form a high‐level theoretical framework.

## 6 CONCLUSION

The present study adds to the results from previous studies by exploring the extent of post‐traumatic growth among Chinese CAPD patients. Education level, employment and self‐perceived stress are factors that were shown to be related to growth in this study. There was not enough evidence to show that self‐perceived burden was related to growth. These findings indicate that PD patients may benefit from stress caused by illness or treatment, provide available social support and improve active coping strategies. More attention should be placed on less educated or currently unemployed patients. Additionally, the impact of the coping strategies on post‐traumatic growth should be highlighted and integrated into targeted interventions.

## CONFLICT OF INTEREST

There are no conflicts of interest to declare.

## AUTHOR CONTRIBUTIONS

All authors listed meet the authorship criteria according to the latest guidelines of the International Committee of Medical Journal Editors, and all authors are in agreement with the manuscript.

## Data Availability

The data that support the findings of this study are available from the corresponding author upon reasonable request.
